# Application of artificial intelligence to pancreatic adenocarcinoma

**DOI:** 10.3389/fonc.2022.960056

**Published:** 2022-07-22

**Authors:** Xi Chen, Ruibiao Fu, Qian Shao, Yan Chen, Qinghuang Ye, Sheng Li, Xiongxiong He, Jinhui Zhu

**Affiliations:** ^1^ Department of General Surgery, Second Affiliated Hospital Zhejiang University School of Medicine, Hangzhou, China; ^2^ Department of Surgical Ward 1, Ningbo Women and Children’s Hospital, Ningbo, China; ^3^ College of Information Engineering, Zhejiang University of Technology, Hangzhou, China

**Keywords:** pancreatic adenocarcinoma (PC), artificial intelligence (AI), machine learning (ML), artificial neural network (ANN), future perspectives

## Abstract

**Background and Objectives:**

Pancreatic cancer (PC) is one of the deadliest cancers worldwide although substantial advancement has been made in its comprehensive treatment. The development of artificial intelligence (AI) technology has allowed its clinical applications to expand remarkably in recent years. Diverse methods and algorithms are employed by AI to extrapolate new data from clinical records to aid in the treatment of PC. In this review, we will summarize AI’s use in several aspects of PC diagnosis and therapy, as well as its limits and potential future research avenues.

**Methods:**

We examine the most recent research on the use of AI in PC. The articles are categorized and examined according to the medical task of their algorithm. Two search engines, PubMed and Google Scholar, were used to screen the articles.

**Results:**

Overall, 66 papers published in 2001 and after were selected. Of the four medical tasks (risk assessment, diagnosis, treatment, and prognosis prediction), diagnosis was the most frequently researched, and retrospective single-center studies were the most prevalent. We found that the different medical tasks and algorithms included in the reviewed studies caused the performance of their models to vary greatly. Deep learning algorithms, on the other hand, produced excellent results in all of the subdivisions studied.

**Conclusions:**

AI is a promising tool for helping PC patients and may contribute to improved patient outcomes. The integration of humans and AI in clinical medicine is still in its infancy and requires the in-depth cooperation of multidisciplinary personnel.

## 1 Introduction

Pancreatic cancer (PC) is one of the deadliest malignancies worldwide. When all tumor stages are included, its 5-year survival rate is 3%–15%, which is the lowest among all cancer types (1, 2). Although pancreatic ductal adenocarcinoma is relatively rare, with an incidence of 8 to 12 cases per 100,000 per year and a 1.3% lifetime risk of developing the disease, the number of cancer deaths caused by PC ranks seventh overall (3). It ranks third in the United States, second only to colon cancer and lung cancer (1). Moreover, the incidence rate of PC has been increasing in recent years, and the mortality rate has also been rising over the past 10 years (1, 4). Therefore, we must actively explore feasible methods to improve the prognosis of patients. Wherever possible, this review focuses on pancreatic adenocarcinoma. However, it should be understood that when the term “pancreatic cancer” is used, the majority of instances are pancreatic ductal adenocarcinomas.

Artificial intelligence (AI) refers to any technique involving the use of a computer system to emulate human behavior (5). Computer vision, convolutional neural networks, and natural language processing have all seen tremendous advancements in data processing and big data technology, which makes AI become a hot spot and help innovate many fields in recent years, including the medical field. There have been some exciting achievements with AI in radiology, pathology, ophthalmology, and dermatology in the medical field. The combination of AI and modern medical treatments is where medical development is headed in the future.

Compared with the subjects mentioned above, the application of AI in the PC field is in its initial stages. However, the existing research results have shown that AI has the ability to optimize the PC diagnostic and therapeutic processes. Our team believes that combining AI with the diagnostic and treatment technology used today may help improve the prognosis of patients. The goals of this study are to summarize AI’s use in various aspects of the diagnosis and treatment of PC cases and also to discuss its limitations and possible future research directions.

## 2 Overview of AI

**Figure 1:**
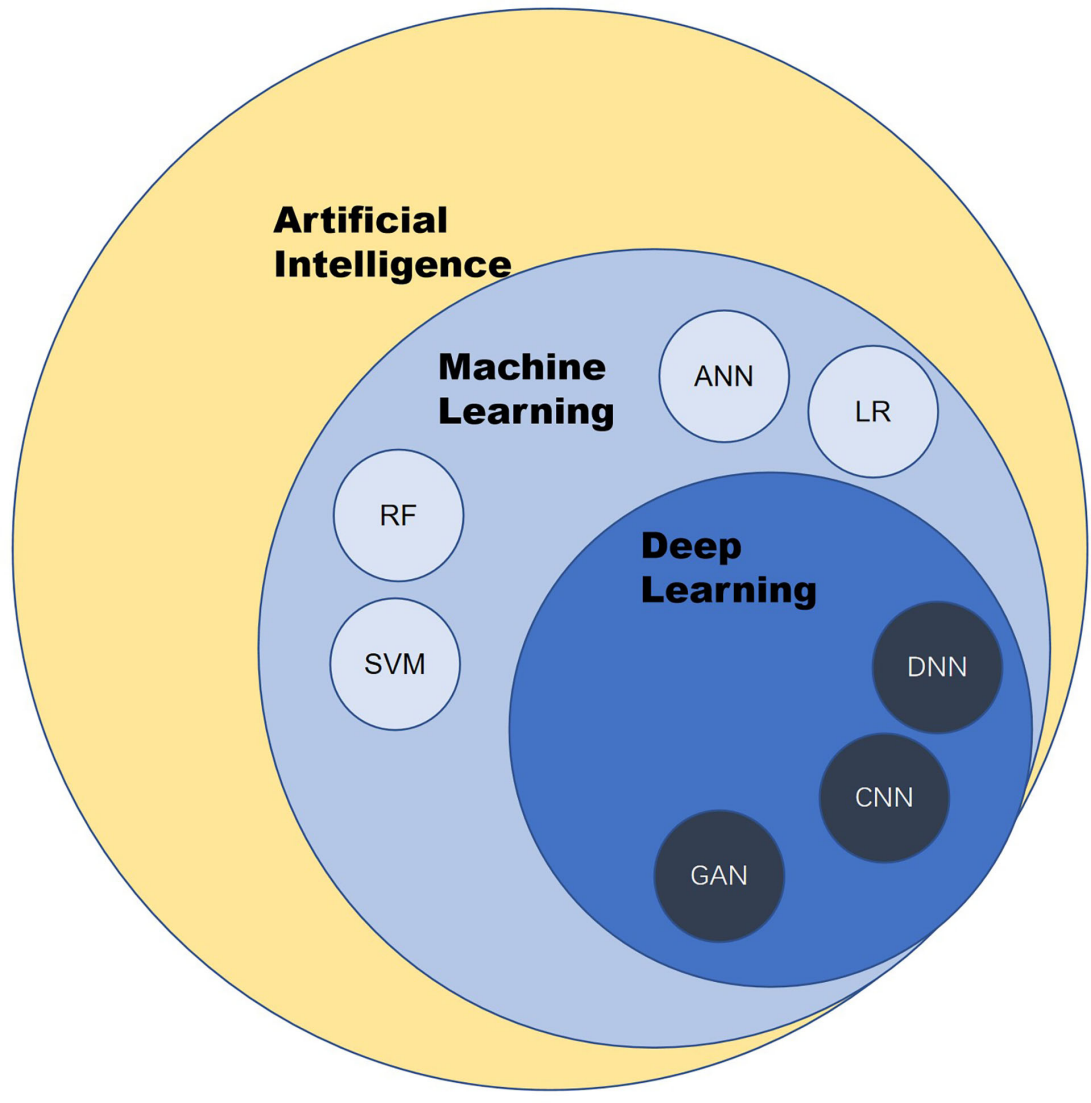
Overview of artificial intelligence. ANN, artificial neural network; CNN, convolutional neural network; LR, logistic regression; RF, random forest; SVM, support vector machine; DNN, deep neural network; GAN, generative adversarial network.

AI is a branch of computer science. It was formally put forward by scientists in 1956. To avoid the question of what “intelligence” is, Alan Turing, the father of AI, tends to test the thinking ability of machines only by comparing the behavior of machines and humans. Given the definition in terms of behavior, AI is a form of technology through which people attempt to use computers to imitate human behavior, especially thinking and decision-making processes (**Figure 1**).

Machine learning (ML) and deep learning are the products of the development of AI. ML is a subset of AI techniques that attempt to apply statistics to data problems to discover new knowledge by generalizing from examples. Deep learning is a subset of ML that uses a collection of sophisticated algorithms known as neural networks to enable machines to analyze and learn in the same way that humans do, allowing them to identify text, pictures, audio, and other input (5).

**Figure 2:**
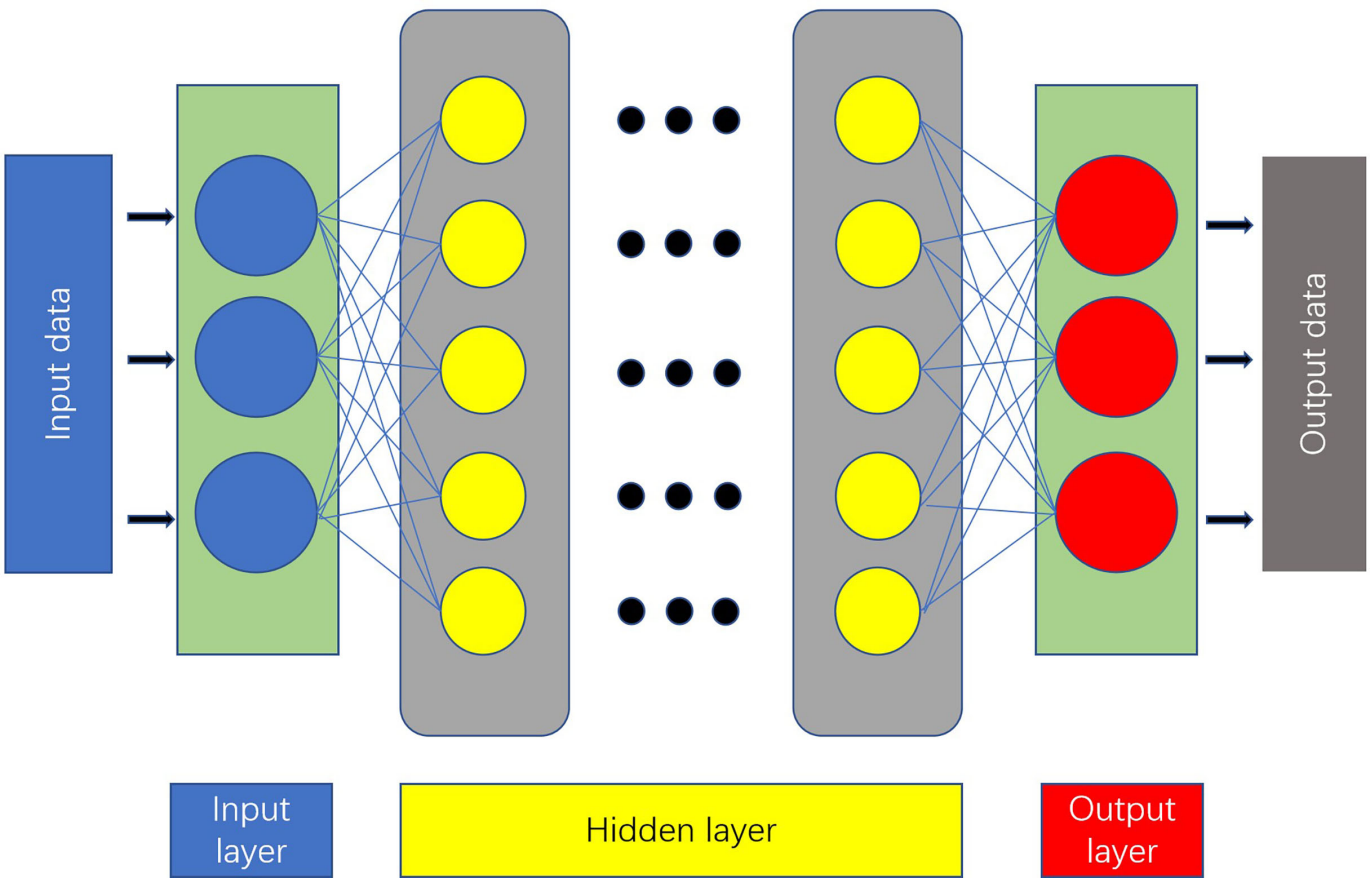
Anatomy of an artificial neural network.

An artificial neural network (ANN) is a computing model made up of interconnected units containing a high number of neurons (6). The most basic ANN consists of three layers (7) (**Figure 2**).

A feedforward neural network with a deep structure and convolution computation is known as a convolutional neural network (8). It has a remarkable capacity to process image information, making it useful for AI technologies.

In general, AI is an information processing technology. In clinical practice, some of the work performed by doctors, such as diagnosing diseases, making treatment plans, and judging prognosis, also involves processing and integrating existing information. Compared with the human brain, a computer has a larger storage space and faster processing speed. Thus, interesting questions have emerged regarding if medical treatment processes such as these can be carried out using AI, as well as if this technology can perform better than humans.

## 3 Methods

We did a thorough analysis of the available literature on AI applications for PC. We searched the online databases PubMed and Google Scholar for publications containing the terms artificial intelligence and pancreatic cancer. Only original articles published with the complete text supplied and written in English were selected using the filter. The abstracts of the publications were then scrutinized for subject relevance. We further looked through the reference lists of pertinent literature reviews to find extra relevant papers.

## 4 Results

According to medical tasks addressed in the research, papers can be grouped into four main categories: risk assessment, diagnosis, treatment, and prognosis prediction.

We will introduce the characteristics of each model and their performance one by one in the following sections. The performance of the models below is generally measured by accuracy, sensitivity, and specificity values and the area under the receiver operating characteristic curve (AUC).

Because each study is conducted in different settings, it is not advisable to compare the performance measurements provided in each study. We highly advise the readers to read each article in order to reach their own judgments.

**Tables 1** and **2** describe some of the technical terminologies that will be mentioned below.

**Table 1:** Performance measure terminologies.

Performance measure	Concept
AUC	AUC is a performance measure typically used in classification problems. The ROC curve consists of a plot of the true-positive rate *vs*. the false-positive rate for the different threshold possibilities. The closer the AUC value is to 1, the better the model classifies.
IoU	The concordance rate between the ground-truth area and the automatic segmentation area was calculated using the intersection over the union (IoU), which is a value ranging from 0 to 1 that is calculated by dividing the area of overlap between the ground-truth area and the automatic segmentation area by the area of union.
DSC	Dice similarity coefficient measures the similarity of the prediction image and the ground-truth image. Its value range is 0–1 and the closer it is to 1, the better the effect of the model.
C-index	Concordance index refers to the proportion of all patient pairs whose predicted results are consistent with the actual results. The value of the C-index is between 0.5 and 1: 0.5 is completely random, indicating that the model has no prediction effect, and 1 is completely consistent, indicating that the prediction result of the model is completely consistent with the actual situation.

AUC, area under the receiver operating characteristic curve; IoU, intersection over the union; DSC, Dice similarity coefficient; C-index, concordance index.

**Table 2:** Technical terminologies.

Technical terminology	Concept
The shadowed set theory	The shadowed set theory is mainly used for data description and data selection. Essential (core) data and boundary data can be automatically obtained with the use of shadowed sets.
CTTA	CT texture analysis is a postprocessing technique that can assess attenuation values and tumor heterogeneity in a user-defined ROI on CT images. CT texture analysis includes parameters that quantify the spatial pattern or arrangement of pixel intensities, as well as CT histogram parameters that characterize the shape of the histogram by using a statistical evaluation of image intensities in the ROI.
The Brennan nomogram score	The Brennan nomogram score is a kind of nomogram that predicts the probability that a patient will survive pancreatic cancer for 1, 2, and 3 years from the time of the initial resection, assuming that there is no death from an alternate cause.
GLRLM	Gray-level run-length matrix provides the size of homogeneous runs for each gray level along a specific linear direction, which is defined by four different directions in the 2D GLRLM, i.e., 0°, 45°, 90°, and 135°. In the GLRLM, the rows are represented by gray values, and the columns are represented by the number of the same adjacent pixels. The gray-level non-uniformity (GLN) features were calculated from the GLRLM matrix for the four directions.
ROI	The region of interest is defined in machine vision and image processing as a box, circle, ellipse, or irregular polygon drawn from the processed picture.

CTTA, computed tomography texture analysis; GLRLM, gray-level run-length matrix; ROI, the region of interest.

### 4.1 Predicting PC through risk factors

There are two types of risk factors for PC: modifiable and non-modifiable risk factors. Smoking, drinking, a history of chronic pancreatitis, dietary variables, and a history of certain infections such as hepatitis B, hepatitis C, and *Helicobacter pylori* are among the modifiable risk factor (9). The latter includes age, gender, ethnicity, blood group, gut bacteria, family history, and genetic susceptibility (9). For these numerous risk factors, there is no standard scale to determine the risk of PC in an individual. This causes clinicians not able to analyze all risk factors together, leading to wasted information and delayed diagnosis.

Prediagnosis symptoms include new-onset diabetes (10, 11), weight loss (12), jaundice, upper abdominal pain, etc., and these often appear a few months to a few years before PC is diagnosed (13). However, because of the non-specificity of these symptoms, doctors are often unable to link them to PC (14, 15). This also leads to wasted information and delayed diagnosis.

One of the reasons for the high mortality rate of PC is its lagging diagnosis. The majority (80%–85%) of people with PC have locally progressed or distant metastasis when they are diagnosed, and only a few patients have tumors that can be surgically removed (15%–20%) (16). AI can help solve this diagnosis delay problem caused by the waste of information. After obtaining the information on the above risk factors from the electronic health records and establishing the corresponding algorithm, this information can be fully used as input for the algorithm. The algorithm then weighs each risk factor and exports the possibility of PC. Some AI algorithms, such as unsupervised ML (17), can even summarize risk factors from electronic health records on their own. Using these algorithms, high-risk groups can be identified and screened to reduce the diagnosis time.

AI has been used in the analysis of electronic health data of a PC patient in several research studies (18–24). The relevant literature can be found in **Table 3**. Most of these studies include AI that analyzes electronic health records to help identify PC high-risk groups months to several years earlier than patients who did not get the help of AI predictions.

**Table 3:** Predicting pancreatic cancer through risk factors.

Ref.	Instrument	No. of patients	Medical task	Performance
Li et al. (18)	ANN	4,361	PDAC prediction	Accuracy of 67.62%
Appelbaum et al. (19)	LR model	594	PDAC prediction	AUC of 0.68
Malhotra et al. (20)	RF model	1,139	PDAC prediction	AUC of 0.609
Muhammad et al. (21)	ANN	898	PDAC prediction	AUC of 0.85
Placido et al. (22)	ANN	24,000	PDAC prediction	AUC of 0.91
Zhao et al. (23)	BNI model	98	PDAC prediction	AUC of 0.910
Hsieh et al. (24)	ANN	1,324,669	NOD predicting PDAC	AUC of 0.727

ANN, artificial neural network; PDAC, pancreatic ductal adenocarcinoma; NOD, new-onset diabetes; AUC, area under the receiver operating characteristic curve; LR, logistic regression; RF, random forest; BNI, Bayesian network inference.

### 4.2 Diagnosis of PC through imaging pictures

The most often utilized imaging procedures in PC diagnosis are computed tomography (CT), magnetic resonance imaging (MRI), and endoscopic ultrasonography (EUS). In a complete diagnostic process, the pancreas image is first obtained using corresponding instruments and then interpreted by radiologists, who then give the final diagnosis. The objectivity of this process will inevitably be affected by the participation of radiologists. Unlike machines, the performance of human brains generally varies, particularly in image recognition. Several circumstances, such as weariness, stress, or a lack of expertise, might cause a lesion to be missed or misdiagnosed. Applying AI can achieve the following objectives: 1) shorten the time spent on image interpretation and improve work efficiency, 2) reduce labor intensity for radiologists, 3) improve the accuracy of diagnosis, and 4) diagnose the disease in an earlier stage and improve patient prognosis.

Compared with research focusing on electronic health records, research on combining AI and imaging is more prevalent and includes more mature technology. In the following, the application of AI in EUS, CT, and MRI will be introduced.

#### 4.2.1 EUS

EUS is substantially better than trans-abdominal ultrasonography (US), CT, or MRI for obtaining high-resolution pictures of the pancreas (25). However, the endoscopist’s expertise and technical proficiency have a significant role in the diagnostic performance of EUS, which affects the objectivity and stability of interpretation, and AI will assist in resolving this issue.

As shown in **Table 4**, some studies reported the application of AI for the analysis of PC EUS images (26–33). Although few studies have been done, it has been claimed that using ML and DL to image the pancreas with EUS can produce results that are on par with or better than those made by endoscopists.

**Table 4:** Diagnosis of pancreatic cancer by endoscopic ultrasonography.

Ref.	AI instrument	Medical task	Patient	Performance
Norton et al. (26)	ANN	PDAC *vs*. CP	21	Accuracy of 89%
Tonozuka et al. (27)	CNN	PDAC *vs*. NP *vs*. CP	76	AUC of 0.940
Saftoiu et al. (28)	ANN	PDAC *vs*. CP	68	Sensitivity of 94.64%
Saftoiu et al. (29)	ANN	PDAC *vs*. CP	258	Accuracy of 84.27%
Saftoiu et al. (30)	ANN	PDAC *vs*. CP	167	Sensitivity of 94.64%
Ozkan et al. (31)	ANN	PDAC *vs*. NP	332	Accuracy of 87.5%
Udristoiu et al. (32)	CNN	PDAC *vs*. PNET *vs*. CPP	30	AUC of 0.98
Marya et al. (33)	CNN	PDAC *vs*. AIP	292	Sensitivity of 91%

ANN, artificial neural network; CNN, convolutional neural network; AUC, area under the receiver operating characteristic curve; PDAC, pancreatic ductal adenocarcinoma; CP, chronic pancreatitis; NP, normal pancreas; CCP, chronic pseudotumoral pancreatitis; PNET, pancreatic neuroendocrine tumor.

Intraductal papillary mucinous neoplasms (IPMNs) are precursor lesions of PC (34). To discriminate between benign and malignant IPMNs, Kuwahara et al. (35) designed a convolutional neural network (CNN), and their model achieved an accuracy of 94.0%. In a similar study, Machicado et al. (36) designed two CNN algorithms to help with IPMN diagnosis and risk classification. Compared with the existing guidelines [the American Gastroenterological Association (AGA) and revised Fukuoka guidelines], both algorithms yielded higher performance for diagnosis.

Zhang et al. (37) explored assistance with interpreting real-time ultrasonograms to help doctors reduce the missed diagnosis rate. Two algorithms were developed: one to help locate the detector and one to help segment the pancreatic region. Using these algorithms, the accuracy of trainee station recognition increased from 67.2% to 78.4%.

The ROI of the above experiments was drawn manually, typically by senior imaging scholars, while some experiments utilized an automated drawing of the ROI. Iwasa et al. (38) evaluated the capability of deep learning for the automatic segmentation of pancreatic tumors on contrast-enhanced endoscopic ultrasound video images. Their algorithm achieved the median intersection over the union of all cases of 0.77.

#### 4.2.2 CT

The most often utilized imaging modality for the first examination of suspicious PC is the CT (39). Up to 34 months before the diagnosis of PDAC, the initial signs of PC, such as pancreatic parenchyma inhomogeneity and loss of typical fatty marbling of the pancreas, have been documented on retrospective CT evaluation (40). These subtle changes are difficult to recognize with the naked eye, which further emphasizes the necessity to possibly implement AI.

There are two phases in using AI for picture analysis of a PC. The initial step is to use the abdominal CT picture to get the contour of the pancreas, a process referred to as segmentation. The second step is to analyze the region generated by segmentation. In the following studies, most of the focus is on the second step, while the first step is usually completed by experienced imaging experts using manual segmentation methods.

##### 4.2.2.1 Research focusing on analyzing the ROI

A summary of the most recent works describing the combination of AI with CT images to diagnose PDAC can be found in **Table 5** (41, 42, 44–46). The performance of these models in the table is satisfying.

**Table 5:** Diagnosis of pancreatic cancer by computerized tomography.

Ref.	Instrument	Patient	Medical task	Performance
Chu et al. (41)	RF	190	PDAC *vs*. NP	AUC of 99.9%
Park et al. (42)	RF	93	PDAC *vs*. AIP	Accuracy of 95.2%
Liu et al. (43)	CNN	238	PDAC *vs*. NP	AUC of 0.92
Ren et al. (44)	LR	79	PDAC *vs*. MFP	AUC of 0.98
Qureshi et al. (45)	NBC	36	PDAC *vs*. NP	Accuracy of 86.0%

AUC, area under the receiver operating characteristic curve; PDAC, pancreatic ductal adenocarcinoma; CP, chronic pancreatitis; NP, normal pancreas; AIP, autoimmune pancreatitis; CNN, convolutional neural network; MFP, mass-forming pancreatitis; LR, logistic regression; RF, random forest; NBC, naive Bayes classifier.

Some experiments have also explored the possibility of using CT images to predict the malignant potential of IPMNs. Qiu et al. (47) employed a support vector machine (SVM) to discriminate different histopathological grades of PDAC. The SVM achieved an overall accuracy of 86%. Hanania et al. (48) conducted a texture analysis of pancreatic images of IPMN patients. Within the gray-level co-occurrence matrix (GLCM), they discovered 14 imaging biomarkers and established corresponding logistic regression models to predict histopathological grade within cyst contours. The best logistic regression yielded an AUC of 0.96. In another similar experiment, Permuth et al. (49) combined a plasma-based miRNA genomic classifier data with radiomic features, and their algorithm revealed an AUC = 0.92.

Due to its strong computing power, AI processes information very quickly. Liu et al. (46) employed a faster region-based convolution network model to accurately read CT images and diagnose PC. Their system was able to acquire medical reports in about 200 ms per picture, which is much less time than imaging professionals take for diagnosis, and the AUC was 0.9632.

##### 4.2.2.2 Research focusing on dividing ROI

Segmentation of the pancreas is often not satisfactory because while comprising just a small proportion of CT pictures, this organ is typically very changeable in form, size, and placement.

The richer feature convolutional network (RCF) is an algorithm used for the automated segmentation of images, but its ability to segment the pancreas is poor. Fu et al. (50) extended the RCF and generated a novel pancreas segmentation network. Finally, their algorithm achieved a 36% Dice similarity coefficient (DSC) value in testing data. Zhou et al. (51) built a fixed-point model that shrank the input region using an anticipated segmentation mask. Their algorithm achieved 82.37% DSC.

Muscle atrophy and decreased muscle density 2 to 4 months after diagnosis were linked to a worse survival rate in individuals with advanced PC (52). However, manual measurements of body composition are too time-consuming. Hsu et al. (53) designed an ANN that was able to quantify the tissue components. The detector analysis took 1 ± 0.5 s and the DSC values for visceral fat, subcutaneous fat, and muscle were 0.80, 0.92, and 0.85, respectively. This algorithm can help doctors save a lot of time while maintaining considerable accuracy.

##### 4.2.2.3 Research focusing on dividing and analyzing the ROI

Chu et al. (54) conducted an experiment that divided PC diagnosis into two steps. Firstly, the algorithm recognized the boundaries of all organs in the abdomen and then identified the PC tissue (54, 55). In the first step, the algorithm’s pancreas segmentation accuracy was 87.8% ± 3.1%. In the second step, the algorithm had 94.1% sensitivity and 98.5% specificity. Liu et al. (56) also proposed a two-stage architecture in which the pancreas was first segmented into a binary mask, then compressed into a shape vector, and anomaly classification was conducted. Finally, they achieved a specificity of 90.2% and a sensitivity of 80.2%. It even picked up on a few difficult instances that radiologists would normally overlook, which showed promise for clinical applications.

#### 4.2.3 MRI

Compared with CT, there are still few studies using MRI images as input data.

Corral et al. (57) employed a deep learning protocol to classify IPMNs. According to the malignant degree of the lesion, IPMNs can be classified as healthy pancreas, low-grade IPMN, or high-grade IPMN with adenocarcinoma. In their algorithm, a whole pancreas image was used as the input, with the malignant degree of the pancreas being the output. Their algorithm’s sensitivity and specificity for detecting dysplasia were 92% and 52%, respectively. Their method has a sensitivity and specificity of 75% and 78%, respectively, for detecting high-grade dysplasia or malignancy. According to this study, deep learning may offer diagnostic accuracy comparable to, if not greater than, existing radiographic recommendations for identifying IPMNs.

Unsupervised learning is the process of solving pattern recognition problems using training samples with unknown categories. Because radiologists are needed to get annotations for the majority of medical imaging operations, obtaining labels to develop machine learning models is time-consuming and costly. Hussein et al. (17) explored an unsupervised learning algorithm. They proposed a new clustering algorithm and tested it for the categorization of IPMNs. The accuracy, sensitivity, and specificity to identify benign or malignant tissues were 58.04%, 58.61%, and 41.67% respectively. Although the performance of their algorithm was not satisfying, this is one of the earliest and largest studies of an IPMN classification computer-aided diagnostic system.

In general, it is difficult to obtain medical images. It leads to a small training set for developing an AI algorithm, negatively affecting its performance. A generative adversarial network (GAN) is an image augmentation technology that can generate high-quality synthetic images from existing ones (58). Gao et al. (59) employed this technique to process their training set and expanded the initial 10,293 patches intercepted from MRI imaging to 35,735. With the help of GAN, they optimized their network.

A three-dimensional (3D) image of a tumor provides vital information about the tumor phenotype and microenvironment. However, making reasonable use of 3D picture information is tough. A 3D neural network, on the one hand, necessitates a significant number of processing resources due to its multiple parameters and complex connections between these parameters. The information in two-dimensional (2D) slices, on the other hand, is insufficient to completely reflect the 3D properties of a tumor. Chen et al. (60) developed a method for the automatic prediction of TP53 mutations in PC. While converting 3D images to 2D images, their spiral transformation technology could reduce the computation for the 3D image but still utilize its information. Finally, their algorithm achieved an AUC of 0.74. Their methods for using 3D information with a small sample size and successful multimodal fusion are possible medical imaging analysis paradigms.

Some research has focused on the automatic segmentation of pancreatic MRI images. Zheng et al. (61) proposed a 2D deep learning-based method to segment such images. Based on the shadowed set theory, the suggested technique defined the uncertain regions of pancreatic MRI images. Finally, they achieved a DSC of 84.37%.

### 4.3 AI for PC treatment

Liu et al. (62) employed a method to help with pancreatic adaptive radiotherapy, which can enhance treatment accuracy and, as a result, reduce gastrointestinal toxicity. They developed this method for cone-beam CT to synthetic CT generation, and the synthetic CT pictures may be able to produce accurate dosage calculations that are equivalent to the planning CT images.

Patients with advanced PC may benefit from echoendoscopic celiac plexus neurolysis as treatment for cancer pain. The efficiency of this procedure is limited; it frequently necessitates repeating therapy, and the outcome of such therapy is not consistent (63, 64). Facciorusso et al. (65) built an ANN model to predict pain response in a patient who underwent repeat echoendoscopic celiac plexus neurolysis (rCPN). They classified the treatment response as effective or ineffective according to the change degree and duration of the visual simulation scale (VAS). Their algorithm achieved an AUC of 0.94. It meant that this algorithm can identify accurately patients likely to benefit from rCPN and exclude those who are not sensitive to the treatment.

Nasief et al. (66) developed a delta-radiomic process for early prediction of PC treatment response based on ML. They analyzed daily CTs recorded during standard CT-guided chemoradiation treatment for PC patients to derive delta-radiomic characteristics. They added a new feature to standard deviation difference termed normalized entropy (NESTD). This new feature can be utilized to enhance organ boundary recognition and offer a method for standardizing contour validation. The output of their model is good or bad response, and their best-performing prediction model achieved an AUC of 0.94.

### 4.4 AI for predicting PC prognosis

PC is among the most lethal cancers. Even if surgery is performed, the estimated survival time is quite limited (67, 68). Additionally, adverse reactions are commonly encountered with surgery, chemotherapy, and radiotherapy (69–71). Therefore, predicting the prognosis of patients according to existing information, measuring the risks and benefits brought by treatment, and then choosing an appropriate treatment scheme are of great importance for improving the health and wellbeing of people. For decision-making, many predictive evaluation methods or risk scores have been established, including perioperative mortality risk (72), postsurgery complications (73), and survival prediction (74, 75). However, the performance of these systems did not meet expectations, and some clinical data were obtained using invasive operations such as surgery. Additional reliable prognostic indicators are urgently needed. The powerful data processing ability of AI gives it the potential to solve these problems. There are studies describing the computer-based quantitative evaluation of tumor morphology on diagnostic imaging in various cancer types, and it is progressively showing promise in describing the underpinning of tumor biology (76–79).

CT texture analysis is a postprocessing technique that can assess attenuation values and tumor heterogeneity in a user-defined ROI on CT images. In patients with non-small cell lung cancer, esophageal cancer, and metastatic renal cell carcinoma, baseline and first posttherapy alterations in CT texture analysis parameters of tumors have been linked to survival (80–82). This technology is used in many of the studies introduced below.

#### 4.4.1 Predicting patient outcomes after the operation

Mu et al. (83) developed a deep learning model based on preoperative CT to anticipate clinically relevant postoperative pancreatic fistula (CR-POPF) following pancreatoduodenectomy and investigate the biological foundations of their model. Within 1–2 min, the model could generate output (with CR-POPF or not) with exceptional performance (AUC of 0.90). In a similar study, Kambakamba et al. (84) and their best classifier “REPTree” achieved an AUC of 0.95.

Lee et al. (85) used AI approaches to analyze the recurrence of PC after surgery. They compared the random forest model with the Cox proportional hazards model. The random forest and Cox model’s C-index averages were 0.68 and 0.77, respectively, in this study. This is the first study to use AI and multicenter registry data to forecast disease-free survival following PC surgery. The results of this methodological investigation show that AI can be a useful decision-support system for patients undergoing PC surgery.

#### 4.4.2 Survival time prediction

Chakraborty et al. (86) quantified the heterogeneity of PDAC in CT images using texture analysis to predict patient 2-year survival rates. The proposed features obtained an AUC of 0.90 using a customized feature selection approach and a naive Bayes classifier.

Tong et al. (87) established ANN models to predict the 8-month survival rates of PC patients with unresectable tumors using clinical factors. Their ANN model with the best result achieved an AUC of 0.92. In a similar study, Walczak et al. (88) developed an ANN model that predicts PC patients’ 7-month survival, and their algorithm achieved 91% sensitivity and 38% specificity.

Integrating mRNA profiling, DNA methylation, and corresponding clinical information together, Tang et al. (89) established CNN models to predict the 5-year survival rates of PC patients, and their best algorithm achieved an AUC of 0.937.

Yue et al. (90) stratified the risks of PC patients by performing a quantitative analysis of pre- and postradiotherapy positron emission tomography-computed tomography (PET-CT) images and determining the predictive usefulness of textural differences in predicting patients’ therapeutic response. Based on the multivariate analytic risk score, the patients were divided into two groups: a low-risk group with a longer mean OS (29.3 months) and a high-risk group with a shorter mean OS (17.7 months). With log-rank *P* = 0.001, the multivariate analysis resulted in substantial risk stratification. In a similar study, Cozzi et al. (91) appraised the ability of a radionics signature that was extracted from CT images to anticipate patient outcomes following stereotactic body radiation (SBRT). The patients were stratified into two groups based on the radiomics signature—a low-risk group with a longer mean OS (14.4 months) and a high-risk group with a shorter mean OS (9.0 months)—and their best model achieved an AUC of 0.73.

Smith et al. (92) offered a unique Bayesian statistical method and used it to predict real lymph node ratio statuses and OS in patients who underwent radical oncologic resection. The predictor variables were obtained from the NCI SEER cancer registry. The C-index for the predictive performance was 0.65, which showed that its accuracy was low. They also developed a web application with a point-and-click interface for entering patient baseline data and seeing the posterior estimated survival statistics, such as median survival time and survival rate, and the LNR from their model.

Kaissis et al. (93) developed a supervised ML algorithm to predict OS in patients with PC by employing diffusion-weighted imaging-derived radiomic features. For the prediction of OS, their algorithm has a sensitivity of 87%, specificity of 80%, and AUC of 90%.

#### 4.4.3 Exploring the factors related to prognosis

We separated the subsequent experiments into three groups based on the treatment received by the test subjects: In the first group, all of the patients underwent surgery. In the second group, none of the patients underwent surgery but received radiotherapy and chemotherapy. In the third group, some patients underwent surgery and some underwent radiotherapy and chemotherapy.

##### 4.4.3.1 The first group

Mucins (MUC) are important in pancreatic tumor development and invasion. Yokoyama et al. (94) built models based on the methylation state of three mucin genes (*MUC1*, *MUC2*, and *MUC4*), and they found that their model outperformed tumor size, lymph node metastasis, distant metastasis, and age in predicting OS and can be used to supplement the TNM staging system’s prognostic value.

Cassinotto et al. (95) assessed the effectiveness of quantitative imaging biomarkers for evaluating pathologic tumor aggressiveness and predicting disease-free survival (DFS) by employing CT texture analysis. They concluded that on CT scans tumors that are more hypoattenuating in the portal venous phase are more likely to be aggressive, with a higher tumor grade, more lymph node invasion, and a shorter DFS.

Attiyeh et al. (96) generated a survival prediction model for resected PDAC patients using preoperative serum cancer antigen 19-9 levels, CT texture features, and the Brennan score. Finally, the concordance index of their model was 0.73. In another similar study, Choi et al. (97) measured texture analysis parameters from T2-weighted images of patients. In their study, following the multivariate Cox analysis, only tumor size continued to be a significant predictor.

Yun et al. (98) conducted a texture analysis of preoperative contrast-enhanced CT imaging of patients undergoing curative resection. They discovered that weaker CT texture analysis scores are linked to a reduced chance of survival. In a similar study, Eilaghi et al. (99) found that longer OS is connected with less inverse difference normalized and greater dissimilarity. Kim et al. (100) analyzed the gray-level non-uniformity (GLN) values of their images, and they found that GLN values were correlated with recurrence-free survival.

Ciaravino et al. (101) assessed how useful CT texture analysis was for evaluating tissue changes in PC that had been shrunk and removed after chemotherapy. At least two CT scans were necessary for the patients in this investigation (one before chemotherapy and one after chemotherapy), and patients received surgical treatment after chemotherapy. They found that the only parameter that was statistically different between CT 1 and CT 2 was kurtosis. In a similar study, Kim et al. (102) found that higher subtracted entropy and lower subtracted GLCM entropy are predictors of a favorable outcome. These two studies showed that patients’ prognoses can be predicted by quantitative analysis of PC pictures before and after chemotherapy.

##### 4.4.3.2 The second group

Sandrasegaran et al. (103) evaluated the efficacy of CT texture analysis (CTTA) in forecasting the prognosis of patients with unresectable PC. The mean value of positive pixels (MPP), kurtosis, entropy, and skewness were selected as the CTTA parameters. In a multivariate Cox proportional hazard analysis, MPP was the only CTTA parameter that showed significance. Additionally, Kaplan–Meier statistics suggested that patients with high MPP and high kurtosis values had worse prognoses. In a similar study, Cheng et al. (104) found that higher standard deviation (SD) values were closely linked to progression-free survival and OS, indicating higher intratumoral heterogeneity. This could help predict better survival outcomes in patients with unresectable PC.

Cui et al. (105) did a quantitative study of PET-CT scans for patients who had locally progressed PC and were receiving SBRT and generated a signature. After multivariate analysis, the suggested signature was shown to be the sole relevant prognostic indicator, scoring 0.66 on the C-index.

##### 4.4.3.3 The third group

Hayward et al. (106) constructed predictive models for the clinical performance of PC patients using ML techniques and compared the results with those of the linear and logistic regression techniques. They concluded that for most target attributes, such as survival time, Bayesian techniques provided the best overall performance.

During the administration of chemoradiation treatment for PC, Chen et al. (107) looked into radiation-induced alterations in quantitative CT characteristics of malignancies and reported that patients with good responses tended to have different texture features (such as volume, skewness, and kurtosis) compared with those with poor tumor responses.

## 5 Discussion

### 5.1 Future perspectives

In the 21st century, computer science is progressing exponentially. This has brought great changes to many other fields, including the medical field. The combination of big data and AI is referred to by some as the fourth industrial revolution (108). Daily diagnosis and treatment activities produce a large variety of medical data, such as imaging, vital signs, laboratory examination results, and more. For physicians, it is difficult to manually integrate all data and conduct a comprehensive analysis. Such situations that involve processing a large amount of data are the strength and advantage of using AI.

For the role of AI in the future, one possibility is that this technology will serve as an assistant to physicians that can provide advice and assist with their final decision-making. This could be very helpful when physicians are fatigued and stressed, and may also contribute to medical education. A second possibility is that AI will fully substitute for a physician. The abovementioned studies demonstrate that AI has the potential to surpass human beings, and in this situation, doctors can be replaced. When AI can completely replace humans in this regard, these individuals may feel liberated to do other work. Moreover, as a kind of software, these technologies can be easily shared worldwide and bring advanced technologies to areas with underdeveloped medical technology.

### 5.2 Challenges

Although the future of AI looks promising, a series of problems need to be addressed to efficiently achieve these goals.

1) The total amount of data is relatively small. The incidence rate of PC is very low (109), which makes it difficult to collect cases. Furthermore, many existing clinical data are not labeled or annotated. As a result, most PDAC experiments are retrospective and single center and have small sample sizes, making them vulnerable to selection bias and recall bias. To address this problem, a multi-agency cooperative model must be established, and prospective, double-blind, multicenter studies can be conducted. This would allow training sets to be representative and improve the performance of AI. Various data augmentation algorithms, such as GAN, can also help solve such problems and increase the amount of the original data.

2) Existing data are not fully utilized. With CT images as an example, most existing studies select the ROI within the section with the largest tumor diameter, resulting in only a 2D section being analyzed. The tumor is a 3D structure, so a 2D section cannot fully represent it. This may be a contributing factor to why the existing models do not perform well. 3D structures are not utilized as input data because ROI selection is done completely manually. The selection of a 2D structure has taken some time (about 3 min per imaging) and would take even longer for a 3D structure. Additionally, 3D structures would provide a very large amount of data. Even a strong computer would need a considerable amount of time to process such data. Therefore, to solve this problem, we need to optimize the algorithm, improve computing power, and develop automatic segmentation of 3D ROIs. Moreover, the existing research often only analyzes imaging results and does not consider the additional data. In fact, we can combine imaging with clinical features (such as weight loss, jaundice, and upper abdominal pain) and laboratory examination (such as CA199) to form hybrid biomarkers to optimize the performance of the model.

3) There is no unified standard for each process involved with obtaining and processing data. For example, standards involved with contrast-enhanced CT images frequently differ, including the type, concentration, and injection speed of the contrast agent; the equipment for obtaining images; scanning parameters; the selection of final images; the format and pixels transformed into analyzable data; and the selection of ROIs. This variability in standards prevents meaningful comparisons between experimental results and makes cooperation among institutions quite difficult. Therefore, experts should establish a reasonable and unified standardized process, which would greatly accelerate the progress of AI in the diagnosis and treatment of PC.

4) The ethical issues involved with medical data also require attention. In most of the abovementioned studies, the data are typically anonymized and the informed consent step is omitted. This would not be an issue in small sample retrospective studies but could be if AI is widely used in clinical practice. AI is an evolving and iterative system that requires the addition of data from new patients in the clinic. While being diagnosed and treated by AI, new patients also expose their data to AI, and the developers of the technology can profit from it. This raises the questions of how benefits should be distributed and if new patients should receive a share.

The storage, sharing, and management of patient data also require urgent attention. Three different models, the centralized, federated, and hybrid models, have been established for data exchange, each with its own set of benefits and drawbacks (see references for details) (110–114). For instance, the biggest disadvantage of the centralized model is that storing information on a server can increase the danger of a security breach because the individual source no longer has control over the data.

5) AI output interpretability is not satisfactory and is referred to as the “black box” (115). The black box model is a system that does not reveal its internal mechanism. In machine learning, black box models describe models that cannot be understood by looking at parameters, such as deep neural networks. People can only directly observe data input and the resulting output from the model, and it is difficult for people to fully understand how the data are being processed. This makes it difficult for people to modify the internal structure of the model to improve the performance of the algorithm. It also makes it difficult for AI to gain the trust of professionals and patients. Some studies (116) employed new technologies, such as the visual analysis approach and eye tracker to increase the interpretability of the model, providing us with a good solution.

6) The process is not automated enough. People’s impression of AI is always linked to full automation, but at this stage, most experiments can only be defined as semiautomatic. Humans often participate in some part of the process. For example, most ROI selections in CT images are manually produced by senior imaging experts. This takes a considerable amount of time, which is contrary to one of the original intentions of using AI. Although some studies have already focused on the segmentation of abdominal organs (50, 51, 54, 55, 61, 117–121), the performance of these algorithms is far below the level of imaging experts. If complete automation is to be achieved, emphasis should be placed on researching accurate organ segmentation methods.

7) The functions of existing AI are too limited. Ideally, AI, like human beings, can interpret pancreatic images to judge the occupied space, the location and nature of this space, and whether surgery is possible. Combined with other clinical information, AI can judge the prognosis of patients. However, AI in the existing research often has only one function. In future studies, we can aim to combine multiple models for AI to be more comprehensive in the PC diagnostic and treatment processes.

### 5.3 Conclusions

AI has the potential to help PC patients and contribute to improved patient outcomes. However, the integration of AI and human intelligence in clinical medicine is still in its infancy. Research to date has reported that the performance of AI could be superior to the standard statistical methods or even humans. Yet, AI still has its limitations, including those that we discussed above. Solving these problems requires the in-depth cooperation of multidisciplinary personnel. In the future, these limitations will be individually addressed, and AI will become an indispensable clinical auxiliary tool.

## Author contributions

All authors listed have made a substantial, direct, and intellectual contribution to the work and approved it for publication.

## Funding

This work was supported by Zhejiang Province Bureau of Health (No. 2020366835) and Funds of Science Technology Department of Zhejiang Province (No. 2020C03074). We thank J. Iacona, Ph.D., from Liwen Bianji (Edanz) (www.liwenbianji.cn) for editing the English text of a draft of this manuscript.

## Conflict of interest

The authors declare that the research was conducted in the absence of any commercial or financial relationships that could be construed as a potential conflict of interest.

## Publisher’s note

All claims expressed in this article are solely those of the authors and do not necessarily represent those of their affiliated organizations, or those of the publisher, the editors and the reviewers. Any product that may be evaluated in this article, or claim that may be made by its manufacturer, is not guaranteed or endorsed by the publisher.
